# *In Vitro* Activity of Delafloxacin against Contemporary Bacterial Pathogens from the United States and Europe, 2014

**DOI:** 10.1128/AAC.02609-16

**Published:** 2017-03-24

**Authors:** M. A. Pfaller, H. S. Sader, P. R. Rhomberg, R. K. Flamm

**Affiliations:** a JMI Laboratories, North Liberty, Iowa, USA; b University of Iowa, Iowa City, Iowa, USA

**Keywords:** MRSA, delafloxacin

## Abstract

The *in vitro* activities of delafloxacin and comparator antimicrobial agents against 6,485 bacterial isolates collected from medical centers in Europe and the United States in 2014 were tested. Delafloxacin was the most potent agent tested against methicillin-susceptible Staphylococcus aureus (MSSA), methicillin-resistant S. aureus, Streptococcus pneumoniae, viridans group streptococci, and beta-hemolytic streptococci and had activity similar to that of ciprofloxacin and levofloxacin against certain members of the Enterobacteriaceae. Overall, the broadest coverage of the tested pathogens (Gram-positive cocci and Gram-negative bacilli) was observed with meropenem and tigecycline in both Europe and the United States. Delafloxacin was shown to be active against organisms that may be encountered in acute bacterial skin and skin structure infections, respiratory infections, and urinary tract infections.

## INTRODUCTION

The fluoroquinolone class of antibiotics is currently used as standard empirical therapy in health care-associated infections and community-acquired infections; specifically, antibiotics of this class are indicated for the treatment of urinary tract infections (UTI), respiratory tract infections (RTI), acute bacterial skin and skin structure infections (ABSSSI), and intra-abdominal infections (1–6). A recent point-prevalence study of antimicrobial use in U.S. acute care hospitals found levofloxacin to be the third most common antimicrobial agent prescribed to treat both community-acquired infections and health care-acquired infections (7). In the face of such broad utilization, the emergence of fluoroquinolone resistance has been observed in both Gram-positive cocci (GPC) and Gram-negative bacilli (GNB) (1, 6, 8).

Fluoroquinolones are the only class of antibiotics in clinical use that directly target two essential bacterial enzymes in DNA replication: DNA gyrase and topoisomerase IV (1, 9). Resistance to fluoroquinolones is primarily caused by target mutations (e.g., mutations in chromosomal genes that encode the subunits of DNA gyrase and topoisomerase IV), efflux pumps, and reduced target expression (9). These mechanisms may occur in various combinations in resistant strains of staphylococci, Pseudomonas aeruginosa, and Enterobacteriaceae (1, 6). Efforts to combat this resistance to the fluoroquinolone class have focused on improving activity against multidrug-resistant bacteria and providing a lower potential for the development of bacterial resistance (1, 4, 5, 8).

Delafloxacin is an anionic investigational fluoroquinolone with documented efficacy in phase 2 trials for the treatment of RTI and ABSSSI and has recently completed phase 3 trials for the treatment of ABSSSI (1, 10). Unlike other quinolones, which usually have a binding affinity for either DNA gyrase or topoisomerase IV, delafloxacin is equally potent against both enzymes (1, 11–13). This dual targeting is believed to help reduce the selection of resistant mutants *in vitro* and *in vivo* (11, 12, 14). Unlike other fluoroquinolones, the mutant prevention concentration for delafloxacin is within 1- to −2-log_2_ dilutions of the MIC value (13). Additionally, the anionic structure of delafloxacin may enhance its potency in acidic environments, characteristic of the milieu at an infection site (1, 13, 15).

Delafloxacin is active *in vitro* against a broad range of Gram-positive and Gram-negative bacteria, including anaerobes and atypical respiratory tract pathogens (e.g., Legionella, Chlamydia, and Mycoplasma) (1, 13, 16–18). Delafloxacin exhibits very low MIC values against Gram-positive pathogens, including fluoroquinolone-resistant strains of Staphylococcus aureus, coagulase-negative staphylococci (CoNS), and Streptococcus pneumoniae (1, 12, 13, 19). It has been shown to be highly active *in vitro* against pathogens that are found in ABSSSI, including methicillin-resistant S. aureus (MRSA), methicillin-resistant coagulase-negative staphylococci (MR-CoNS), beta-hemolytic streptococci, Enterobacteriaceae, P. aeruginosa, and anaerobes (10, 11, 13, 16, 20). Delafloxacin is also active against bacteria associated with RTIs, including S. pneumoniae, Haemophilus influenzae, and Moraxella catarrhalis (19).

The aim of the present study was to examine the susceptibility profiles of delafloxacin and comparator agents when tested against contemporary clinical isolates collected from European and U.S. medical centers during surveillance year 2014.

## RESULTS AND DISCUSSION

### Overall activity of delafloxacin.

The MIC distributions for select organisms or organism groups from U.S. and European medical centers are shown in Table 1. The MIC_90_ values for U.S. and European isolates of GPC were within ±1 log_2_ dilution step for each organism group except methicillin-susceptible S. aureus (MSSA) (MIC_90_s, 0.03 and ≤0.004 μg/ml for U.S. and European isolates, respectively) and methicillin-susceptible coagulase-negative staphylococci (MS-CoNS; MIC_90_s, 0.12 and 0.008 μg/ml for U.S. and European isolates, respectively) (data not shown).

**TABLE 1:** Cumulative frequency distribution of delafloxacin in MIC results for Europe and the United States^*a*^

Organism or organism group	No. (%) of isolates for which MIC (μg/ml) was:													MIC_50_ (μg/ml)	MIC_90_ (μg/ml)
	Total	0.004	0.008	0.015	0.03	0.06	0.12	0.25	0.5	1	2	4	>4		
Staphylococcus aureus															
US	1,100	666 (60.5)	8 (61.3)	8 (62.0)	38 (65.5)	183 (82.1)	62 (87.7)	63 (93.5)	24 (95.6)	34 (98.7)	0 (98.7)	14 (100.0)	0 (100.0)	≤0.004	0.25
EU	250	193 (77.2)	2 (78.0)	1 (78.4)	4 (80.0)	12 (84.8)	13 (90.0)	18 (97.2)	3 (98.4)	3 (99.6)	0 (99.6)	1 (100.0)	0 (100.0)	≤0.004	0.12
MSSA															
US	591	515 (87.1)	7 (88.3)	4 (89.0)	10 (90.7)	27 (95.3)	11 (97.1)	8 (98.5)	6 (99.5)	2 (99.8)	0 (99.8)	1 (100.0)	0 (100.0)	≤0.004	0.03
EU	186	176 (94.6)	2 (95.7)	1 (96.2)	2 (97.3)	1 (97.8)	2 (98.9)	1 (99.5)	0 (99.5)	1 (100.0)			0 (100.0)	≤0.004	≤0.004
MRSA															
US	509	151 (29.7)	1 (29.9)	4 (30.6)	28 (36.1)	156 (66.8)	51 (76.8)	55 (87.6)	18 (91.2)	32 (97.4)	0 (97.4)	13 (100.0)	0 (100.0)	0.06	0.5
EU	64	17 (26.6)	0 (26.6)	0 (26.6)	2 (29.7)	11 (46.9)	11 (64.1)	17 (90.6)	3 (95.3)	2 (98.4)	0 (98.4)	1 (100.0)	0 (100.0)	0.12	0.25
Coagulase-negative staphylococci															
US	100	51 (51.0)	7 (58.0)	3 (61.0)	1 (62.0)	6 (68.0)	14 (82.0)	4 (86.0)	4 (90.0)	9 (99.0)	1 (100.0)		0 (100.0)	≤0.004	0.5
EU	100	43 (43.0)	8 (51.0)	1 (52.0)	6 (58.0)	10 (68.0)	10 (78.0)	14 (92.0)	5 (97.0)	3 (100.0)			0 (100.0)	0.008	0.25
Enterococcus faecalis															
US	300	0 (0.0)	2 (0.7)	0 (0.7)	28 (10.0)	150 (60.0)	39 (73.0)	9 (76.0)	27 (85.0)	37 (97.3)	8 (100.0)		0 (100.0)	0.06	1
EU	150	2 (1.3)	2 (2.7)	0 (2.7)	16 (13.3)	57 (51.3)	31 (72.0)	3 (74.0)	17 (85.3)	22 (100.0)			0 (100.0)	0.06	1
Enterococcus faecium															
US	195		0 (0.0)	1 (0.5)	0 (0.5)	8 (4.6)	0 (4.6)	1 (5.1)	6 (8.2)	8 (12.3)	3 (13.8)	16 (22.1)	152 (100.0)	>4	>4
EU	100	0 (0.0)	1 (1.0)	1 (2.0)	0 (2.0)	0 (2.0)	0 (2.0)	2 (4.0)	1 (5.0)	2 (7.0)	1 (8.0)	7 (15.0)	85 (100.0)	>4	>4
Streptococcus pneumoniae															
US	300	34 (11.3)	169 (67.7)	84 (95.7)	7 (98.0)	3 (99.0)	2 (99.7)	1 (100.0)					0 (100.0)	0.008	0.015
EU	150	16 (10.7)	90 (70.7)	40 (97.3)	4 (100.0)								0 (100.0)	0.008	0.015
Viridans group streptococci															
US	196	34 (17.3)	41 (38.3)	71 (74.5)	34 (91.8)	7 (95.4)	4 (97.4)	2 (98.5)	1 (99.0)	1 (99.5)	1 (100.0)		0 (100.0)	0.015	0.03
EU	98	19 (19.4)	19 (38.8)	30 (69.4)	18 (87.8)	8 (95.9)	0 (95.9)	3 (99.0)	1 (100.0)				0 (100.0)	0.015	0.06
Streptococcus pyogenes															
US	283	67 (23.7)	170 (83.7)	46 (100.0)									0 (100.0)	0.008	0.015
EU	150	33 (22.0)	94 (84.7)	20 (98.0)	3 (100.0)								0 (100.0)	0.008	0.015
Streptococcus agalactiae															
US	150	18 (12.0)	70 (58.7)	56 (96.0)	3 (98.0)	0 (98.0)	1 (98.7)	1 (99.3)	1 (100.0)				0 (100.0)	0.008	0.015
EU	75	5 (6.7)	38 (57.3)	28 (94.7)	1 (96.0)	1 (97.3)	1 (98.7)	1 (100.0)					0 (100.0)	0.008	0.015
Streptococcus dysgalactiae															
US	82	19 (23.2)	51 (85.4)	11 (98.8)	1 (100.0)								0 (100.0)	0.008	0.015
EU	50	18 (36.0)	30 (96.0)	1 (98.0)	1 (100.0)								0 (100.0)	0.008	0.008
Enterobacteriaceae															
US	1,500	3 (0.2)	16 (1.3)	132 (10.1)	261 (27.5)	389 (53.4)	163 (64.3)	74 (69.2)	94 (75.5)	102 (82.3)	116 (90.0)	70 (94.7)	80 (100.0)	0.06	2
EU	750	1 (0.1)	11 (1.6)	81 (12.4)	118 (28.1)	196 (54.3)	76 (64.4)	25 (67.7)	30 (71.7)	49 (78.3)	64 (86.8)	44 (92.7)	55 (100.0)	0.06	4
Escherichia coli															
US	300	2 (0.7)	11 (4.3)	77 (30.0)	71 (53.7)	14 (58.3)	13 (62.7)	7 (65.0)	3 (66.0)	11 (69.7)	34 (81.0)	34 (92.3)	23 (100.0)	0.03	4
EU	200	1 (0.5)	9 (5.0)	61 (35.5)	43 (57.0)	14 (64.0)	8 (68.0)	9 (72.5)	2 (73.5)	6 (76.5)	24 (88.5)	16 (96.5)	7 (100.0)	0.03	4
E. coli isolates of the ESBL phenotype															
US	52	0 (0.0)	1 (1.9)	2 (5.8)	2 (9.6)	2 (13.5)	1 (15.4)	1 (17.3)	1 (19.2)	4 (26.9)	17 (59.6)	14 (86.5)	7 (100.0)	2	>4
EU	40		0 (0.0)	3 (7.5)	4 (17.5)	0 (17.5)	1 (20.0)	2 (25.0)	0 (25.0)	2 (30.0)	11 (57.5)	11 (85.0)	6 (100.0)	2	>4
Klebsiella pneumoniae															
US	225		0 (0.0)	2 (0.9)	30 (14.2)	108 (62.2)	25 (73.3)	11 (78.2)	11 (83.1)	6 (85.8)	4 (87.6)	11 (92.4)	17 (100.0)	0.06	4
EU	164		0 (0.0)	1 (0.6)	12 (7.9)	64 (47.0)	14 (55.5)	2 (56.7)	5 (59.8)	7 (64.0)	10 (70.1)	17 (80.5)	32 (100.0)	0.12	>4
K. pneumoniae isolates of the ESBL phenotype															
US	35				0 (0.0)	1 (2.9)	1 (5.7)	0 (5.7)	2 (11.4)	4 (22.9)	2 (28.6)	9 (54.3)	16 (100.0)	4	>4
EU	67				0 (0.0)	4 (6.0)	2 (9.0)	0 (9.0)	1 (10.4)	4 (16.4)	8 (28.4)	17 (53.7)	31 (100.0)	4	>4
Klebsiella oxytoca															
US	75			0 (0.0)	3 (4.0)	44 (62.7)	23 (93.3)	3 (97.3)	2 (100.0)				0 (100.0)	0.06	0.12
EU	36			0 (0.0)	4 (11.1)	18 (61.1)	11 (91.7)	1 (94.4)	1 (97.2)	1 (100.0)			0 (100.0)	0.06	0.12
Pseudomonas aeruginosa															
US	100		0 (0.0)	1 (1.0)	1 (2.0)	7 (9.0)	26 (35.0)	22 (57.0)	8 (65.0)	10 (75.0)	7 (82.0)	6 (88.0)	12 (100.0)	0.25	>4
EU	100			0 (0.0)	1 (1.0)	3 (4.0)	22 (26.0)	28 (54.0)	11 (65.0)	8 (73.0)	1 (74.0)	7 (81.0)	19 (100.0)	0.25	>4
Acinetobacter baumannii-A. calcoaceticus															
US	100		0 (0.0)	1 (1.0)	10 (11.0)	21 (32.0)	11 (43.0)	4 (47.0)	7 (54.0)	5 (59.0)	8 (67.0)	8 (75.0)	25 (100.0)	0.5	>4
EU	100			0 (0.0)	3 (3.0)	8 (11.0)	5 (16.0)	1 (17.0)	5 (22.0)	7 (29.0)	18 (47.0)	27 (74.0)	26 (100.0)	4	>4

a Abbreviations: EU, Europe; US, United States; MSSA, methicillin-susceptible S. aureus; MRSA, methicillin-resistant S. aureus; ESBL, extended-spectrum β-lactamase.

Delafloxacin showed very low MICs against Gram-positive pathogens (Table 1). Among the S. aureus isolates, 99.5% of MSSA isolates from both U.S. and European study sites were inhibited at the pharmacodynamic breakpoint of ≤0.5 μg/ml (1, 13, 21). European isolates of MRSA, MS-CoNS, and MR-CoNS were slightly more susceptible to delafloxacin than U.S. isolates at an MIC of ≤0.5 μg/ml (for MRSA isolates, 95.3 and 91.2% isolates from Europe and the United States, respectively; for MS-CoNS isolates, 100.0 and 97.6% isolates from Europe and the United States, respectively; and for MR-CoNS isolates, 95.5 and 84.5% isolates from Europe and the United States, respectively) (data not shown). Notably, among fluoroquinolone-resistant (FQ^r^) strains of S. aureus and CoNS, 88.3% (484/548) were inhibited by ≤0.5 μg/ml of delafloxacin (data not shown).

The potency of delafloxacin against U.S. and European isolates of enterococci and streptococci was similar (Table 1). Delafloxacin was most active against isolates of S. pneumoniae and beta-hemolytic streptococci (MIC_50_ and MIC_90_, 0.008 and 0.015 μg/ml, respectively, for each group of organisms) and viridans group streptococci (MIC_50_ and MIC_90_, 0.015 and 0.03 μg/ml, respectively). All FQ^r^ strains of S. pneumoniae (5/5) were inhibited by ≤0.25 μg/ml of delafloxacin. The MIC_50_ and MIC_90_ against U.S. and European isolates of E. faecalis were 0.06 and 1 μg/ml, respectively, whereas isolates of Enterococcus faecium were not susceptible to delafloxacin (Table 1).

Similar to the activity of delafloxacin against GPC, the activity of delafloxacin was comparable against isolates of GNB from the United States and Europe, with the exception of Enterobacter spp. (MIC_90_s, 0.5 μg/ml for U.S. isolates and 2 μg/ml for European isolates), Providencia spp. (MIC_90_s, >4 μg/ml for U.S. isolates and 1 μg/ml for European isolates), other Enterobacteriaceae (MIC_90_s, 0.5 μg/ml for U.S. isolates and 0.12 μg/ml for European isolates), and Acinetobacter baumannii-A. calcoaceticus (MIC_50_, 0.5 μg/ml for U.S. isolates and 4 μg/ml for European isolates). Delafloxacin was most active against Klebsiella oxytoca (MIC_50_ and MIC_90_, 0.06 and 0.12 μg/ml, respectively), Enterobacter aerogenes (MIC_50_ and MIC_90_, 0.12 and 0.25 μg/ml, respectively), Citrobacter koseri (MIC_50_ and MIC_90_, 0.015 and 0.06 μg/ml, respectively), and other Enterobacteriaceae (MIC_50_ and MIC_90_, 0.06 and 0.25 μg/ml, respectively) and was the least active against Klebsiella pneumoniae, Providencia spp., P. aeruginosa, and Acinetobacter baumannii-A. calcoaceticus (MIC_90_s, >4 μg/ml for all isolates) (Table 1). The activity of delafloxacin was considerably greater against strains of E. coli of the non-extended-spectrum β-lactamase [ESBL]-producing phenotype (non-ESBL phenotype) than strains of E. coli of the ESBL-producing phenotype (ESBL phenotype) (MIC_50_, 0.03 μg/ml versus 2 μg/ml, respectively), non-ESBL-phenotype and ESBL-phenotype strains of K. pneumoniae (MIC_50_, 0.06 μg/ml versus 4 μg/ml, respectively), and non-ESBL-phenotype and ESBL-phenotype strains of P. mirabilis (MIC_50_, 0.06 μg/ml versus 2 μg/ml, respectively). Delafloxacin retained potent activity against ESBL-phenotype strains of K. oxytoca (MIC_50_ and MIC_90_, 0.06 and 0.12 μg/ml, respectively) and was more active against ceftazidime-susceptible than ceftazidime-nonsusceptible strains of P. aeruginosa (MIC_50_, 0.25 μg/ml versus 4 μg/ml, respectively). More than 90% of FQ^r^ GNB showed decreased susceptibility (MIC, ≥2 μg/ml) to delafloxacin.

### Susceptibilities of European and U.S. Gram-positive isolates to delafloxacin and comparator agents.

The activities of delafloxacin and comparator agents tested against European (250 isolates) and U.S. (1,100 isolates) isolates of S. aureus are shown in Table 2. Delafloxacin was the most potent antimicrobial agent tested against isolates of MSSA (MIC_50_ and MIC_90_, ≤0.004 and 0.008 μg/ml, respectively) and on the basis of the MIC_90_s was 8- to at least 64-fold more potent than ceftaroline and at least 64-fold more potent than levofloxacin (Table 2). Tigecycline (MIC_50_ and MIC_90_, 0.06 and 0.06 μg/ml, respectively), delafloxacin (MIC_50_ and MIC_90_, 0.06 and 0.5 μg/ml, respectively), and daptomycin (MIC_50_ and MIC_90_, 0.25 and 0.5 μg/ml, respectively) were the most potent agents tested against MRSA (Table 2). Delafloxacin was at least 64-fold more potent than levofloxacin (according to the MIC_50_s) and at least 8-fold more potent than ceftaroline against MRSA. MRSA strains exhibited high levels of resistance against levofloxacin (68.9 and 68.9% according to Clinical and Laboratory Standards Institute [CLSI] and European Committee on Antimicrobial Susceptibility Testing [EUCAST] criteria, respectively) and erythromycin (79.9 and 83.8% according to CLSI and EUCAST criteria, respectively) (Table 2). The greatest coverage of all S. aureus isolates (MSSA and MRSA isolates from both Europe and the United States) was provided by linezolid, tigecycline, and vancomycin (to which 100.0% of isolates were susceptible). Isolates from both Europe and United States also exhibited high levels of susceptibility to daptomycin (99.8% of isolates were susceptible), ceftaroline (98.0%), and trimethoprim-sulfamethoxazole (98.5%) (Table 2).

**TABLE 2:** Activities of delafloxacin and comparator antimicrobial agents when tested against U.S. and European Gram-positive isolates

Organism group (no. of isolates tested)/antimicrobial agent	% of isolates susceptible by the following criteria:		MIC (μg/ml)		
	CLSI	EUCAST	50%	90%	Range
Staphylococcus aureus (1,350)					
Delafloxacin			≤0.004	0.25	≤0.004 to 4
Levofloxacin	64.4	64.4	0.25	>4	≤0.12 to >4
Ceftaroline	98.0	98.0	0.25	1	0.03 to 2
Ciprofloxacin	0.0	0.0	64	>128	64 to >128
Clindamycin	87.0	86.8	≤0.25	>2	≤0.25 to >2
Daptomycin	99.8	99.8	0.25	0.5	≤0.06 to 2
Erythromycin	45.9	46.3	4	>16	≤0.12 to >16
Linezolid	100.0	100.0	1	1	0.25 to 2
Oxacillin	57.6	57.6	0.5	>2	≤0.25 to >2
Tetracycline	94.3	92.5	≤0.5	≤0.5	≤0.5 to >8
Tigecycline	100.0^*a*^	100.0	0.06	0.06	≤0.015 to 0.5
Trimethoprim-sulfamethoxazole	98.5	98.5	≤0.5	≤0.5	≤0.5 to >4
Vancomycin	100.0	100.0	1	1	0.25 to 2
MSSA (777)					
Delafloxacin			≤0.004	0.008	≤0.004 to 4
Levofloxacin	89.8	89.8	0.25	2	≤0.12 to >4
Ceftaroline	100.0	100.0	0.25	0.25	0.03 to 1
Ciprofloxacin	0.0	0.0	>128	>128	>128 to >128
Clindamycin	94.0	93.7	≤0.25	≤0.25	≤0.25 to >2
Daptomycin	100.0	100.0	0.25	0.5	≤0.06 to 1
Erythromycin	69.6	69.8	0.25	>16	≤0.12 to >16
Linezolid	100.0	100.0	1	1	0.25 to 2
Oxacillin	100.0	100.0	0.5	0.5	≤0.25 to 2
Tetracycline	95.9	94.2	≤0.5	≤0.5	≤0.5 to >8
Tigecycline	100.0^*a*^	100.0	0.06	0.06	≤0.015 to 0.5
Trimethoprim-sulfamethoxazole	99.0	99.0	≤0.5	≤0.5	≤0.5 to >4
Vancomycin	100.0	100.0	1	1	0.25 to 2
MRSA (573)					
Delafloxacin			0.06	0.5	≤0.004 to 4
Levofloxacin	30.0	30.0	4	>4	≤0.12 to >4
Ceftaroline	95.3	95.3	1	1	0.25 to 2
Ciprofloxacin	0.0	0.0	>128	>128	64 to >128
Clindamycin	77.5	77.5	≤0.25	>2	≤0.25 to >2
Daptomycin	99.5	99.5	0.25	0.5	0.12 to 2
Erythromycin	13.8	14.3	>16	>16	≤0.12 to >16
Linezolid	100.0	100.0	1	1	0.25 to 2
Oxacillin	0.0	0.0	>2	>2	>2 to >2
Tetracycline	92.1	90.2	≤0.5	1	≤0.5 to >8
Tigecycline	100.0^*a*^	100.0	0.06	0.06	≤0.015 to 0.5
Trimethoprim-sulfamethoxazole	97.9	97.9	≤0.5	≤0.5	≤0.5 to >4
Vancomycin	100.0	100.0	1	1	0.5 to 2
MS-CoNS (75)					
Delafloxacin			≤0.004	0.06	≤0.004 to 1
Levofloxacin	88.0	88.0	0.25	4	≤0.12 to >4
Ceftaroline			0.12	0.25	0.03 to 0.5
Clindamycin	84.0	84.0	≤0.25	>2	≤0.25 to >2
Daptomycin	100.0	100.0	0.25	0.5	≤0.06 to 1
Erythromycin	69.3	69.3	≤0.12	>16	≤0.12 to >16
Linezolid	100.0	100.0	0.5	0.5	0.25to1
Oxacillin	100.0	100.0	≤0.25	1	≤0.25 to 2
Tetracycline	89.3	86.7	≤0.5	8	≤0.5 to >8
Tigecycline		100.0	0.03	0.06	≤0.015 to 0.12
Trimethoprim-sulfamethoxazole	92.0	92.0	≤0.5	≤0.5	≤0.5 to >4
Vancomycin	100.0	100.0	1	2	0.25 to 4
MR-CoNS (125)					
Delafloxacin			0.06	0.5	≤0.004 to 2
Levofloxacin	38.4	38.4	4	>4	≤0.12 to >4
Ceftaroline			0.5	1	0.06 to 2
Clindamycin	70.4	67.2	≤0.25	>2	≤0.25 to >2
Daptomycin	99.2	99.2	0.5	0.5	≤0.06 to 2
Erythromycin	25.6	25.6	>16	>16	≤0.12 to >16
Linezolid	100.0	100.0	0.5	0.5	≤0.12 to 1
Oxacillin	0.0	0.0	>2	>2	0.5 to >2
Tetracycline	80.8	77.6	1	>8	≤0.5 to >8
Tigecycline		100.0	0.06	0.12	≤0.015 to 0.25
Trimethoprim-sulfamethoxazole	65.6	65.6	≤0.5	>4	≤0.5 to >4
Vancomycin	100.0	100.0	1	2	0.5 to 2
Enterococcus faecalis (450)					
Delafloxacin			0.06	1	≤0.004 to 2
Levofloxacin	70.7	70.7^*b*^	1	>4	0.25 to >4
Ampicillin	100.0	99.6	1	2	≤0.25 to 8
Ceftaroline			2	8	0.25 to >32
Clindamycin			>2	>2	≤0.25 to >2
Daptomycin	100.0		1	2	0.12 to 4
Erythromycin	4.7		>16	>16	≤0.12 to >16
Linezolid	99.8	100.0	1	1	≤0.12 to 4
Teicoplanin	97.8	97.6	≤2	≤2	≤2 to >16
Tetracycline	23.1		>8	>8	≤0.5 to >8
Trimethoprim-sulfamethoxazole			≤0.5	≤0.5	≤0.5 to >4
Vancomycin	97.8	97.8	1	2	0.5 to >16
Enterococcus faecium (295)					
Delafloxacin			>4	>4	0.008 to >4
Levofloxacin	7.8	10.8^*b*^	>4	>4	0.5 to >4
Ampicillin	10.8	10.8	>8	>8	≤0.25 to >8
Ceftaroline			>32	>32	0.12 to >32
Clindamycin			>2	>2	≤0.25 to >2
Daptomycin	99.0		2	4	0.12 to 8
Erythromycin	3.7		>16	>16	≤0.12 to >16
Linezolid	99.0	100.0	1	1	0.25 to 4
Teicoplanin	47.1	46.1	16	>16	≤2 to >16
Tetracycline	33.2		>8	>8	≤0.5 to >8
Trimethoprim-sulfamethoxazole			≤0.5	>4	≤0.5 to >4
Vancomycin	43.4	43.4	>16	>16	0.25 to >16
Streptococcus pneumoniae (450)					
Delafloxacin			0.008	0.015	≤0.004 to 0.25
Levofloxacin	98.9	98.9	1	1	0.5 to >4
Amoxicillin-clavulanic acid	91.1		≤1	2	≤1 to >8
Ceftaroline	99.6^*c*^	99.3	≤0.015	0.12	≤0.015 to 1
Ceftriaxone	83.6,^*d*^ 94.2^*c*^	83.6	≤0.06	1	≤0.06 to 8
Clindamycin	84.7	84.9	≤0.25	>2	≤0.25 to >2
Erythromycin	59.9	59.9	≤0.12	>16	≤0.12 to >16
Meropenem	84.4	84.4,^*d*^ 100.0^*c*^	≤0.015	0.5	≤0.015 to 2
Moxifloxacin	98.9	98.7	≤0.12	0.25	≤0.12 to 2
Penicillin	63.8,^*e*^ 63.8,^*f*^ 94.4^*g*^	63.8,^*d*^ 63.8^*g*^	≤0.06	2	≤0.06 to 8
Tetracycline	78.4	78.4	≤0.5	>8	≤0.5 to >8
Trimethoprim-sulfamethoxazole	68.9	75.3	≤0.5	>4	≤0.5 to >4
Viridans group streptococci (294)					
Delafloxacin			0.015	0.03	≤0.004 to 2
Levofloxacin	94.1		1	2	≤0.12 to >4
Amoxicillin-clavulanic acid		79.7	≤1	2	≤1 to >8
Ceftaroline			0.03	0.12	≤0.015 to 1
Ceftriaxone	90.9	86.4	0.25	1	≤0.06 to >8
Clindamycin	89.5	89.9	≤0.25	>2	≤0.25 to >2
Erythromycin	53.0		≤0.12	8	≤0.12 to >16
Meropenem	93.7	99.0	0.06	0.25	≤0.015 to 4
Moxifloxacin			≤0.12	0.25	≤0.12 to >4
Penicillin	73.1	79.7	≤0.06	1	≤0.06 to >8
Tetracycline	64.3		≤0.5	>8	≤0.5 to >8
Trimethoprim-sulfamethoxazole			≤0.5	4	≤0.5 to >4
Streptococcus pyogenes (433)					
Delafloxacin			0.008	0.015	≤0.004 to 0.03
Levofloxacin	99.8	96.5	0.5	1	0.25 to >4
Amoxicillin-clavulanic acid	100.0	100.0	≤1	≤1	≤1 to ≤1
Ceftaroline	100.0	100.0	≤0.015	≤0.015	≤0.015 to 0.03
Ceftriaxone	100.0	100.0	≤0.06	≤0.06	≤0.06 to 0.5
Clindamycin	91.5	91.9	≤0.25	≤0.25	≤0.25 to >2
Erythromycin	85.2	85.2	≤0.12	>16	≤0.12 to >16
Meropenem	100.0	100.0	≤0.015	≤0.015	≤0.015 to 0.12
Moxifloxacin		100.0	≤0.12	0.25	≤0.12 to 0.5
Penicillin	100.0	100.0	≤0.06	≤0.06	≤0.06 to 0.12
Tetracycline	80.2	78.6	≤0.5	>8	≤0.5 to >8
Vancomycin	100.0	100.0	0.25	0.5	≤0.12 to 0.5
Streptococcus agalactiae (225)					
Delafloxacin			0.008	0.015	≤0.004 to 0.5
Levofloxacin	97.8	96.9	0.5	1	0.25 to >4
Amoxicillin-clavulanic acid	100.0	100.0	≤1	≤1	≤1 to ≤1
Ceftaroline	100.0	100.0	≤0.015	0.03	≤0.015 to 0.03
Ceftriaxone	100.0	100.0	≤0.06	0.12	≤0.06 to 0.25
Clindamycin	70.7	72.4	≤0.25	>2	≤0.25 to >2
Erythromycin	52.4	52.4	≤0.12	>16	≤0.12 to >16
Meropenem	100.0	100.0	0.03	0.06	≤0.015 to 0.12
Moxifloxacin		97.8	≤0.12	0.25	≤0.12 to >4
Penicillin	100.0	100.0	≤0.06	≤0.06	≤0.06 to ≤0.06
Tetracycline	17.4	17.0	>8	>8	≤0.5 to >8
Vancomycin	100.0	100.0	0.5	0.5	0.25 to 1
Streptococcus dysgalactiae (132)					
Delafloxacin			0.008	0.015	≤0.004 to 0.03
Levofloxacin	99.2	97.0	0.5	1	0.25 to >4
Amoxicillin-clavulanic acid	100.0	100.0	≤1	≤1	≤1 to ≤1
Ceftaroline	100.0	100.0	≤0.015	≤0.015	≤0.015 to ≤0.015
Ceftriaxone	100.0	100.0	≤0.06	≤0.06	≤0.06 to 0.5
Clindamycin	88.6	90.2	≤0.25	0.5	≤0.25 to >2
Erythromycin	68.9	68.9	≤0.12	>16	≤0.12 to >16
Meropenem	100.0	100.0	≤0.015	≤0.015	≤0.015 to 0.06
Moxifloxacin		100.0	≤0.12	0.25	≤0.12 to 0.25
Penicillin	100.0	100.0	≤0.06	≤0.06	≤0.06 to ≤0.06
Tetracycline	61.8	59.5	≤0.5	>8	≤0.5 to >8
Vancomycin	100.0	100.0	0.25	0.25	≤0.12 to 1

a Breakpoints from FDA package insert, revised December 2014.

b Uncomplicated UTI only.

c Using nonmeningitis breakpoints.

d Using meningitis breakpoints.

e Using oral breakpoints.

f Using parenteral, meningitis breakpoints.

g Using parenteral, nonmeningitis breakpoints.

The delafloxacin MIC_50_ and MIC_90_ values for all coagulase-negative staphylococci (CoNS) were 0.008 and 0.5 μg/ml, respectively (Table 1). Tigecycline (MIC_50_ and MIC_90_, 0.03 and 0.06 μg/ml, respectively) and delafloxacin (MIC_50_ and MIC_90_, ≤0.004 and 0.06 μg/ml, respectively) were the most potent agents tested against MS-CoNS (Table 2). When delafloxacin was tested against isolates of MS-CoNS, it was 4-fold more potent than ceftaroline, 8-fold more potent than linezolid, 32-fold more potent than vancomycin, and >64-fold more potent than levofloxacin (according to the MIC_90_s). European isolates of MS-CoNS were more susceptible than U.S. isolates to levofloxacin (97.0% versus 81.0%, respectively), clindamycin (90.9% versus 78.6%, respectively), erythromycin (72.7% versus 66.7%, respectively), tetracycline (93.9% versus 85.7%, respectively), and trimethoprim-sulfamethoxazole (100.0% versus 85.7%, respectively) (data not shown).

The antibiogram results for MR-CoNS isolates from both Europe (67 isolates) and the United States (58 isolates) showed higher MIC values for all tested drugs except daptomycin (to which 99.2% of isolates were susceptible), linezolid (to which 100.0% of isolates were susceptible), and vancomycin (to which 100.0% of isolates were susceptible). Tigecycline (MIC_50_ and MIC_90_, 0.06 and 0.12 μg/ml, respectively), delafloxacin (MIC_50_ and MIC_90_, 0.06 and 0.5 μg/ml, respectively), linezolid (MIC_50_ and MIC_90_, 0.5 and 0.5 μg/ml, respectively), and ceftaroline (MIC_50_ and MIC_90_, 0.5 and 1 μg/ml, respectively) were the most potent antimicrobials tested against both European and U.S. strains of MR-CoNS. Levofloxacin, clindamycin, erythromycin, and trimethoprim-sulfamethoxazole all showed limited activity against MR-CoNS isolates from both regions.

All isolates of E. faecalis from Europe and the United States were susceptible to ampicillin (Table 2). A small number of E. faecalis strains were resistant to vancomycin (2.2%). Delafloxacin (MIC_50_ and MIC_90_, 0.06 and 1 μg/ml, respectively) and linezolid (MIC_50_ and MIC_90_, 1 and 1 μg/ml, respectively) were the most potent antimicrobials tested (Table 2).

Delafloxacin (MIC_50_ and MIC_90_, >4 and >4 μg/ml, respectively; 10.5% of isolates were susceptible to delafloxacin at ≤1 μg/ml), levofloxacin (MIC_50_ and MIC_90_, >4 and >4 μg/ml, respectively; 7.8 and 10.8% of isolates were susceptible according to CLSI and EUCAST criteria, respectively), erythromycin (MIC_50_ and MIC_90_, >16 and >16 μg/ml, respectively; 3.7% of isolates were susceptible according to the CLSI criterion), and ampicillin (MIC_50_ and MIC_90_, >8 and >8 μg/ml, respectively; 10.8 and 10.8% of isolates were susceptible according to CLSI and EUCAST criteria, respectively) displayed limited activity against E. faecium strains regardless of geographic region or vancomycin susceptibility patterns (Table 2).

Delafloxacin was the most active agent tested against S. pneumoniae isolates from Europe and the United States (MIC_50_ and MIC_90_, 0.008 and 0.015 μg/ml, respectively) (Table 2). All European isolates (100.0%) and 98.0% of U.S. isolates were inhibited by ≤0.03 μg/ml of delafloxacin; the highest delafloxacin MIC value for U.S. isolates was 0.25 μg/ml (Table 1 and 2). Delafloxacin was 8-fold more active than ceftaroline (MIC_50_ and MIC_90_, ≤0.015 and 0.12 μg/ml, respectively), 16-fold more active than moxifloxacin (MIC_50_ and MIC_90_, ≤0.12 and 0.25 μg/ml, respectively), and 64-fold more active than levofloxacin (MIC_50_ and MIC_90_, 1 and 1 μg/ml, respectively) (Table 2). For other common-use antimicrobials, the rate of penicillin resistance (MIC, ≥2 μg/ml, oral breakpoint) was 11.3% (0.7%; MIC, ≥8 μg/ml, parenteral, nonmeningitis breakpoint), the rate of erythromycin resistance was 39.0%, the rate of tetracycline resistance was 21.6%, and the rate of trimethoprim-sulfamethoxazole resistance was 18.9% (Table 2). The delafloxacin MIC for the three high-level penicillin-resistant (MIC, >4 μg/ml) strains was 0.008 μg/ml (data not shown).

The most active agents tested against the viridans group streptococci were delafloxacin (MIC_50_ and MIC_90_, 0.015 and 0.03 μg/ml; Tables 1 and 2), moxifloxacin (MIC_50_ and MIC_90_, ≤0.12 and 0.25 μg/ml, respectively), and ceftaroline (MIC_50_ and MIC_90_, 0.03 and 0.12 μg/ml, respectively) (Table 2). The rate of resistance to penicillin and ceftriaxone was higher among European isolates (11.2% and 12.2%, respectively) than U.S. isolates (2.6 and 3.1%, respectively). The rates of resistance to levofloxacin and erythromycin were comparable for European isolates (5.1% and 44.9%, respectively) and U.S. isolates (5.6% and 44.6%, respectively) (Table 2). Meropenem exhibited the highest coverage against viridans group streptococci and was more active against U.S. isolates (96.4 and 100.0% of isolates were susceptible according to CLSI and EUCAST criteria, respectively) than European isolates (88.8 and 96.9% of isolates were susceptible according to CLSI and EUCAST criteria, respectively).

The activities of delafloxacin and comparator antimicrobial agents against a total of 790 isolates of beta-hemolytic streptococci (433 isolates of Streptococcus pyogenes, 225 of Streptococcus agalactiae, and 132 of Streptococcus dysgalactiae) were tested (Tables 1 and 2). Delafloxacin was highly potent against these organisms (Table 1). All delafloxacin MIC values for S. pyogenes and S. dysgalactiae were ≤0.03 μg/ml. The highest delafloxacin MIC value for S. agalactiae was 0.5 μg/ml, and 97.3% of S. agalactiae isolates were inhibited by delafloxacin at ≤0.03 μg/ml (Table 1). All beta-hemolytic streptococcal isolates were susceptible to ceftaroline, ceftriaxone, meropenem, penicillin, and vancomycin (Table 2). The rates of resistance to levofloxacin were 0.2% for S. pyogenes, 2.2% for S. agalactiae, and 0.8% for S. dysgalactiae (Table 2). The rate of resistance to erythromycin was higher among isolates of S. agalactiae (46.7%) and S. dysgalactiae (29.5%) than among isolates of S. pyogenes (14.1%). The rate of resistance to clindamycin among isolates of beta-hemolytic streptococci ranged from 8.1% to 27.6% (Table 2).

### Susceptibilities of European and U.S. Gram-negative isolates to delafloxacin and comparator agents.

Delafloxacin was active against the majority of the Enterobacteriaceae, exhibiting MIC_50_ and MIC_90_ values of 0.06 and 4 μg/ml, respectively, and with 80.9% of isolates being inhibited by delafloxacin at ≤1 μg/ml (Table 1). The rates of susceptibility to fluoroquinolones, as measured by the use of ciprofloxacin and levofloxacin, for the Enterobacteriaceae were 81.6% and 83.8%, respectively (Table 3). More than 90% of FQ^r^ Enterobacteriaceae isolates showed decreased susceptibility (MIC, >1 μg/ml) to delafloxacin (data not shown). The rates of susceptibility to aztreonam, ceftriaxone, cefepime, and ceftazidime ranged from 80.3% to 90.8% (Table 3). Meropenem (MIC_50_ and MIC_90_, 0.03 and 0.06 μg/ml, respectively; 97.5 and 97.9% of isolates were susceptible according to CLSI and EUCAST criteria, respectively) and tigecycline (MIC_50_ and MIC_90_, 0.25 and 1 μg/ml, respectively; 99.2 and 95.2% of isolates were susceptible according to CLSI and EUCAST criteria, respectively) were the most active agents (Table 3).

**TABLE 3:** Activity of delafloxacin and comparator antimicrobial agents when tested against U.S. and European Gram-negative isolates

Organism group (no. of isolates tested)/antimicrobial agent	% of isolates susceptible by the following criteria:		MIC (μg/ml)		
	CLSI	EUCAST	50%	90%	Range
Enterobacteriaceae (2,250)					
Delafloxacin			0.06	4	≤0.004 to >4
Levofloxacin	83.8	81.9	≤0.12	>4	≤0.12 to >4
Ampicillin-sulbactam	47.4	47.4	16	>32	0.5 to >32
Aztreonam	86.3	83.6	≤0.12	>16	≤0.12 to >16
Cefepime	90.8	87.8	≤0.5	2	≤0.5 to >16
Ceftazidime	86.3	82.8	0.25	16	0.03 to >32
Ceftriaxone	80.3	80.3	0.12	>8	≤0.06 to >8
Ciprofloxacin	81.6	79.3	≤0.03	>4	≤0.03 to >4
Gentamicin	90.7	89.0	≤1	4	≤1 to >8
Meropenem	97.5	97.9	0.03	0.06	≤0.015 to >32
Piperacillin-tazobactam	89.3	85.7	2	32	≤0.5 to >64
Tigecycline	99.2^*b*^	95.2	0.25	1	0.03 to 4
Escherichia coli (500)					
Delafloxacin			0.03	4	≤0.004 to >4
Levofloxacin	69.6	69.6	≤0.12	>4	≤0.12 to >4
Ampicillin-sulbactam	49.6	49.6	16	>32	0.5 to >32
Aztreonam	86.4	82.6	≤0.12	16	≤0.12 to >16
Cefepime	87.0	84.2	≤0.5	8	≤0.5 to >16
Ceftazidime	89.2	83.4	0.12	8	0.03 to >32
Ceftriaxone	84.0	84.0	≤0.06	>8	≤0.06 to >8
Ciprofloxacin	69.4	68.8	≤0.03	>4	≤0.03 to >4
Gentamicin	86.4	86.0	≤1	>8	≤1 to >8
Meropenem	99.6	99.6	≤0.015	0.03	≤0.015 to 4
Piperacillin-tazobactam	94.2	90.0	2	8	≤0.5 to >64
Tigecycline	100.0^*b*^	100.0	0.06	0.12	0.03 to 1
E. coli isolates of the ESBL phenotype (92)					
Delafloxacin			2	>4	0.008 to >4
Levofloxacin	21.7	21.7	>4	>4	≤0.12 to >4
Ampicillin-sulbactam	16.3	16.3	32	>32	2 to >32
Aztreonam	26.1	5.4	>16	>16	≤0.12 to >16
Cefepime	31.5	20.7	16	>16	≤0.5 to >16
Ceftazidime	41.3	9.8	8	32	0.06 to >32
Ceftriaxone	13.0	13.0	>8	>8	0.25 to >8
Ciprofloxacin	20.7	19.6	>4	>4	≤0.03 to >4
Gentamicin	63.0	62.0	≤1	>8	≤1 to >8
Meropenem	97.8	97.8	≤0.015	0.03	≤0.015 to 4
Piperacillin-tazobactam	81.5	65.2	8	>64	1 to >64
Tigecycline	100.0^*b*^	100.0	0.12	0.12	0.06 to 0.5
Klebsiella pneumoniae (389)					
Delafloxacin			0.06	>4	0.015 to >4
Levofloxacin	81.5	80.2	≤0.12	>4	≤0.12 to >4
Ampicillin-sulbactam	63.2	63.2	8	>32	1 to >32
Aztreonam	77.1	75.8	≤0.12	>16	≤0.12 to >16
Cefepime	77.9^*a*^	75.3	≤0.5	>16	≤0.5 to >16
Ceftazidime	76.9	74.8	0.12	>32	0.03 to >32
Ceftriaxone	75.3	75.3	≤0.06	>8	≤0.06 to >8
Ciprofloxacin	77.4	75.6	≤0.03	>4	≤0.03 to >4
Gentamicin	86.4	85.1	≤1	>8	≤1 to >8
Meropenem	90.2	91.0	0.03	1	≤0.015 to >32
Piperacillin-tazobactam	81.2	75.8	4	>64	≤0.5 to >64
Tigecycline	99.7^*b*^	97.7	0.25	0.5	0.06 to 4
K. pneumoniae isolates of the ESBL phenotype (102)					
Delafloxacin			4	>4	0.06 to >4
Levofloxacin	34.3	32.4	>4	>4	≤0.12 to >4
Ampicillin-sulbactam	1.0	1.0	>32	>32	4 to >32
Aztreonam	12.7	7.8	>16	>16	≤0.12 to >16
Cefepime	15.7	5.9	>16	>16	≤0.5 to >16
Ceftazidime	11.8	3.9	>32	>32	0.25 to >32
Ceftriaxone	5.9	5.9	>8	>8	0.12 to >8
Ciprofloxacin	18.6	15.7	>4	>4	≤0.03 to >4
Gentamicin	48.0	43.1	>8	>8	≤1 to >8
Meropenem	62.7	65.7	0.06	>32	≤0.015 to >32
Piperacillin-tazobactam	31.4	23.5	>64	>64	2 to >64
Tigecycline	99.0^*b*^	96.1	0.25	0.5	0.12 to 4
Klebsiella oxytoca (111)					
Delafloxacin			0.06	0.12	0.03 to 1
Levofloxacin	100.0	100.0	≤0.12	≤0.12	≤0.12 to 1
Ampicillin-sulbactam	63.1	63.1	8	>32	2 to >32
Aztreonam	83.8	81.1	0.25	>16	≤0.12 to >16
Cefepime	98.2^*a*^	96.4	≤0.5	1	≤0.5 to >16
Ceftazidime	98.2	97.3	0.12	0.5	0.03 to >32
Ceftriaxone	82.9	82.9	0.12	>8	≤0.06 to >8
Ciprofloxacin	98.2	98.2	≤0.03	0.06	≤0.03 to 4
Gentamicin	99.1	99.1	≤1	≤1	≤1 to >8
Meropenem	100.0	100.0	0.03	0.03	≤0.015 to 0.06
Piperacillin-tazobactam	81.1	78.4	2	>64	≤0.5 to >64
Tigecycline	100.0^*b*^	100.0	0.12	0.25	0.06 to 1
Proteus mirabilis (211)					
Delafloxacin			0.06	2	0.015 to >4
Levofloxacin	78.7	71.1	≤0.12	>4	≤0.12 to >4
Ampicillin-sulbactam	86.7	86.7	2	16	0.5 to >32
Aztreonam	99.5	98.1	≤0.12	≤0.12	≤0.12 to 8
Cefepime	97.2^*a*^	96.7	≤0.5	≤0.5	≤0.5 to >16
Ceftazidime	97.2	94.3	0.06	0.12	0.03 to 32
Ceftriaxone	93.4	93.4	≤0.06	≤0.06	≤0.06 to >8
Ciprofloxacin	71.6	67.8	≤0.03	>4	≤0.03 to >4
Gentamicin	88.6	85.3	≤1	8	≤1 to >8
Meropenem	100.0	100.0	0.06	0.12	≤0.015 to 1
Piperacillin-tazobactam	100.0	100.0	≤0.5	1	≤0.5 to 8
Tigecycline	94.3^*b*^	64.5	1	2	0.12 to 4
Enterobacter spp. (384)					
Delafloxacin			0.06	1	≤0.004 to >4
Levofloxacin	96.6	95.8	≤0.12	0.5	≤0.12 to >4
Ampicillin-sulbactam	24.1	24.1	32	>32	0.5 to >32
Aztreonam	76.6	73.7	≤0.12	>16	≤0.12 to >16
Cefepime	93.7^*a*^	85.6	≤0.5	2	≤0.5 to >16
Ceftazidime	75.7	73.0	0.25	>32	0.03 to >32
Ceftriaxone	70.6	70.6	0.25	>8	≤0.06 to >8
Ciprofloxacin	95.5	94.5	≤0.03	0.25	≤0.03 to >4
Gentamicin	96.9	96.9	≤1	≤1	≤1 to >8
Meropenem	97.9	99.0	0.03	0.06	≤0.015 to >32
Piperacillin-tazobactam	81.2	77.2	2	64	≤0.5 to >64
Tigecycline	100.0^*b*^	97.6	0.25	0.25	0.03 to 2
Citrobacter spp. (178)					
Delafloxacin			0.06	2	0.008 to >4
Levofloxacin	93.8	92.7	≤0.12	0.5	≤0.12 to >4
Ampicillin-sulbactam	68.5	68.5	4	>32	1 to >32
Aztreonam	89.3	87.1	≤0.12	16	≤0.12 to >16
Cefepime	97.2	94.9	≤0.5	≤0.5	≤0.5 to >16
Ceftazidime	87.6	86.0	0.25	16	0.06 to >32
Ceftriaxone	87.1	87.1	0.12	>8	≤0.06 to >8
Ciprofloxacin	92.1	91.0	≤0.03	0.5	≤0.03 to >4
Gentamicin	95.5	94.4	≤1	≤1	≤1 to >8
Meropenem	97.8	98.3	≤0.015	0.03	≤0.015 to 8
Piperacillin-tazobactam	90.4	85.4	2	16	≤0.5 to >64
Tigecycline	100.0^*b*^	99.4	0.12	0.25	0.06 to 2
Indole-positive Proteus spp. (249)					
Delafloxacin			0.12	4	0.008 to >4
Levofloxacin	75.2	70.0	≤0.12	>4	≤0.12 to >4
Ampicillin-sulbactam	29.6	29.6	16	32	0.5 to >32
Aztreonam	96.0	92.0	≤0.12	1	≤0.12 to >16
Cefepime	95.2^*a*^	94.0	≤0.5	≤0.5	≤0.5 to >16
Ceftazidime	87.2	82.0	0.12	16	0.03 to >32
Ceftriaxone	75.6	75.6	≤0.06	8	≤0.06 to >8
Ciprofloxacin	73.6	66.8	≤0.03	>4	≤0.03 to >4
Gentamicin	85.8	77.6	≤1	8	≤1 to >8
Meropenem	100.0	100.0	0.06	0.12	≤0.015 to 1
Piperacillin-tazobactam	95.2	94.0	≤0.5	4	≤0.5 to >64
Tigecycline	98.4^*b*^	95.2	0.5	1	0.12 to 4
Serratia spp. (193)					
Delafloxacin			1	2	0.03 to >4
Levofloxacin	95.9	93.3	≤0.12	1	≤0.12 to >4
Ampicillin-sulbactam	5.2	5.2	>32	>32	4 to >32
Aztreonam	94.3	90.7	≤0.12	1	≤0.12 to >16
Cefepime	96.4^*a*^	94.8	≤0.5	≤0.5	≤0.5 to >16
Ceftazidime	96.4	93.3	0.25	1	0.03 to >32
Ceftriaxone	84.5	84.5	0.25	4	≤0.06 to >8
Ciprofloxacin	93.8	87.6	0.12	1	≤0.03 to >4
Gentamicin	96.4	94.3	≤1	2	≤1 to >8
Meropenem	97.3	97.9	0.03	0.06	≤0.015 to 8
Piperacillin-tazobactam	92.7	88.1	2	16	≤0.5 to >64
Tigecycline	99.0^*b*^	98.4	0.5	0.5	0.06 to 4
Pseudomonas aeruginosa (200)					
Delafloxacin			0.25	>4	0.015 to >4
Levofloxacin	72.5	62.5	0.5	>4	≤0.12 to >4
Amikacin	93.5	89.5	2	16	≤0.25 to >32
Aztreonam	55.5	3.5	8	>16	0.25 to >16
Cefepime	83.0	83.0	2	16	≤0.5 to >16
Ceftazidime	78.5	78.5	2	>32	0.25 to >32
Ceftriaxone			>8	>8	1 to >8
Ciprofloxacin	75.0	70.0	0.25	>4	≤0.03 to >4
Colistin	98.5	100.0	2	2	≤0.5 to 4
Gentamicin	85.5	85.5	≤1	>8	≤1 to >8
Meropenem	74.4	74.4	0.5	8	≤0.015 to >32
Piperacillin-tazobactam	78.0	78.0	8	>64	≤0.5 to >64
Acinetobacter baumannii-A. calcoaceticus (200)					
Delafloxacin			2	>4	0.015 to >4
Levofloxacin	34.0	33.0	>4>4		≤0.12 to >4
Amikacin	53.5	51.0	8	>32	1 to >32
Ampicillin-sulbactam	40.2		16	>32	0.5 to >32
Aztreonam			>16	>16	4 to >16
Cefepime	36.0		>16	>16	≤0.5 to >16
Ceftazidime	38.5		>32	>32	0.5 to >32
Ciprofloxacin	32.5	32.5	>4	>4	0.06 to >4
Colistin	92.0	92.0	1	2	≤0.5 to >8
Gentamicin	48.0	48.0	8	>8	≤1 to >8
Meropenem	41.2	41.2	16	>32	0.06 to >32
Piperacillin-tazobactam	35.2		>64	>64	≤0.5 to >64

a Intermediate is interpreted as susceptible-dose dependent.

b Breakpoints from the FDA package insert, revised December 2014.

Among ESBL-phenotype isolates of E. coli and K. pneumoniae, the potencies of all comparator agents were markedly decreased (Table 3). Meropenem (97.8 and 97.8% of isolates were susceptible according to CLSI and EUCAST criteria, respectively) retained potent activity against ESBL-phenotype strains of E. coli, whereas the rate of meropenem resistance was high (34.3 and 26.5% of isolates were susceptible according to CLSI and EUCAST criteria, respectively) among isolates of ESBL-producing K. pneumoniae (Table 3). ESBL-phenotype K. pneumoniae isolates remained susceptible to tigecycline (99.0 and 96.1% of isolates were susceptible according to CLSI and EUCAST criteria, respectively). Only 28.3% of ESBL-phenotype E. coli isolates and 18.6% of ESBL-phenotype K. pneumoniae isolates were inhibited by delafloxacin at ≤1 μg/ml (Table 1).

In contrast to the results observed with K. pneumoniae, the activity of delafloxacin was higher against K. oxytoca isolates (100.0% of K. oxytoca isolates but only 76.6% of K. pneumoniae isolates were inhibited by delafloxacin at ≤1 μg/ml; Table 1), including ESBL-phenotype strains (Table 1). The rates of susceptibility to ciprofloxacin, levofloxacin, cefepime, meropenem, gentamicin, and tigecycline for K. oxytoca were >96.0% (Table 3), despite the inclusion of 22 ESBL-phenotype isolates.

Delafloxacin was active against species of Enterobacteriaceae with high rates of ceftazidime resistance due to AmpC β-lactamase production, including Enterobacter, Citrobacter, and Serratia isolates (Tables 1 and 3). Delafloxacin at ≤1 μg/ml inhibited 91.4% of Enterobacter spp. (87.4 and 92.7% of isolates from Europe and the United States, respectively). Delafloxacin MIC values were ≤1 μg/ml for 87.6% of Citrobacter spp. (88.3 and 87.3% of isolates from Europe and the United States, respectively) and 76.7% of Serratia spp. (73.8 and 78.0% of isolates from Europe and the United States, respectively) (Table 1). The rates of susceptibility of isolates of these three genera to ciprofloxacin, levofloxacin, cefepime, meropenem, and tigecycline were >90.0% (Table 3). Proteus mirabilis and indole-positive Proteae were generally susceptible to aztreonam, cefepime, meropenem, and piperacillin-tazobactam but showed decreased susceptibility to the fluoroquinolones, including delafloxacin.

Among European and U.S. isolates of P. aeruginosa, only amikacin (93.5 and 89.5% of isolates were susceptible according to CLSI and EUCAST criteria, respectively) and colistin (98.5 and 100.0% of isolates were susceptible according to CLSI and EUCAST criteria, respectively) were active against >90% of isolates tested (Table 3). Delafloxacin at ≤1 μg/ml inhibited 74.0% of P. aeruginosa isolates (Table 1). The rates of susceptibility to ciprofloxacin were 75.0 and 70.0% according to CLSI and EUCAST criteria, respectively, and the rates of susceptibility to levofloxacin were 72.5 and 62.5% according to CLSI and EUCAST criteria, respectively. Among 40 levofloxacin-resistant isolates of P. aeruginosa, delafloxacin MIC values were >1 μg/ml for 39 isolates (data not shown). The rates of resistance to ceftazidime among isolates of P. aeruginosa were 16.5 and 21.5% according to CLSI and EUCAST criteria, respectively (Table 3). The susceptibility of ceftazidime-resistant P. aeruginosa isolates to all agents except colistin was poor (data not shown).

A. baumannii-A. calcoaceticus isolates were nonsusceptible (intermediate or resistant by CLSI and EUCAST criteria) to most agents tested (Table 3). Delafloxacin at ≤1 μg/ml inhibited 44.0% of isolates (Table 1). The rates of susceptibility to ciprofloxacin and levofloxacin were 32.5% and 34.0%, respectively (Table 3), and ranged from 48.0% to 50.0% for U.S. isolates and from 17.0% to 18.0% for European isolates (data not shown). Only the rate of susceptibility to colistin (MIC_50_ and MIC_90_, 1 and 2 μg/ml, respectively; 92.0 and 92.0% of isolates were susceptible according to CLSI and EUCAST criteria, respectively) achieved a value of >90.0% (Table 3). In general, resistance to the tested agents was greater for European isolates than U.S. isolates of Acinetobacter.

Antibiotic resistance is a growing problem in both European and U.S. medical centers (22). Active surveillance and antimicrobial stewardship efforts are essential to combat this threat to patient safety across all health care settings (23, 24). In the present survey, we examined the *in vitro* susceptibility profiles of 6,485 isolates of GPC and GNB from European and U.S. medical centers for the year 2014. The data from the present survey document the comparable activity of delafloxacin against European and U.S. bacterial isolates. Overall, the broadest coverage of the tested pathogens was observed with meropenem and tigecycline in both Europe and the United States (Tables 2 and 3). The most active agents against staphylococci and streptococci were delafloxacin, daptomycin, and tigecycline, whereas meropenem and tigecycline were the most active agents against GNB. Delafloxacin was active against MRSA, MR-CoNS, viridans group streptococci, beta-hemolytic streptococci, and penicillin- and macrolide-resistant S. pneumoniae strains (Tables 1 and 2). Isolates of E. faecium, ESBL-phenotype Enterobacteriaceae, ceftazidime-nonsusceptible P. aeruginosa, and Acinetobacter were considerably less susceptible to delafloxacin than the GPC and wild-type GNB. In contrast, delafloxacin showed activity comparable to that of the other fluoroquinolones tested against AmpC-producing strains of Enterobacteriaceae.

These data build on reports by previous investigators (11, 12, 14, 19, 20) and indicate that delafloxacin merits further study for the treatment of ABSSSI, RTI, and urinary tract infections where an acid environment and mixed GPC and GNB infections are common.

## MATERIALS AND METHODS

### Organisms.

A total of 6,485 nonduplicate bacterial isolates were collected prospectively from 69 medical centers located in the United States (4,410 isolates) and from 44 medical centers located in 25 European countries (2,075 isolates) in the year 2014. All organisms were isolated from hospitalized patients with bloodstream infections (1,373 isolates), RTI (1,368 isolates), ABSSSI (2,177 isolates), UTI (735 isolates), intra-abdominal infections (267 isolates), and other types of infections (565 isolates). Isolates were identified to the species level at each participating medical center, and the identity was confirmed by the monitoring laboratory (JMI Laboratories, North Liberty, IA, USA) using standard bacteriological algorithms and methodologies or matrix-assisted laser desorption ionization–time of flight mass spectrometry (Bruker, Billerica, MA, USA), when necessary.

### Antimicrobial susceptibility testing.

MICs were determined using the reference Clinical and Laboratory Standards Institute (CLSI) broth microdilution method (25). Quality control (QC) and interpretation of results were performed in accordance with the CLSI M100-S26 standard (26) and the European Committee on Antimicrobial Susceptibility Testing (EUCAST) 2016 guidelines (27). Escherichia coli, Klebsiella pneumoniae, Klebsiella oxytoca, and Proteus mirabilis were grouped as ESBL-phenotype strains on the basis of the CLSI screening criteria for potential ESBL production (i.e., a ceftazidime, ceftriaxone, or aztreonam MIC of ≥2 μg/ml) (26). Isolates of P. aeruginosa were classified as ceftazidime susceptible (MIC, ≤ 8 μg/ml) and ceftazidime nonsusceptible (MICs, >8 μg/ml). QC strains were tested concurrently and included E. coli ATCC 25922 and ATCC 35218, S. aureus ATCC 29213, P. aeruginosa ATCC 27853, Enterococcus faecalis ATCC 29212, and S. pneumoniae ATCC 49619. All QC results were within published ranges.
